# Silencing PTEN in the fallopian tube promotes enrichment of cancer stem cell-like function through loss of PAX2

**DOI:** 10.1038/s41419-021-03663-2

**Published:** 2021-04-07

**Authors:** Angela Russo, Jose A. Colina, Junlone Moy, Seth Baligod, Austin A. Czarnecki, Peter Varughese, Daniel D. Lantvit, Matthew J. Dean, Joanna E. Burdette

**Affiliations:** 1 Department of Pharmaceutical Sciences, University of Illinois at Chicago, Chicago, IL 60607 USA; 2 Department of Animal Sciences, University of Illinois Urbana-Champaign, Urbana, IL 61801 USA

**Keywords:** Cancer, Oncogenesis

## Abstract

High-grade serous ovarian cancer (HGSOC) is the most lethal gynecological malignancy that is primarily detected at the metastatic stage. Most HGSOC originates from the fallopian tube epithelium (FTE) and metastasizes to the ovary before invading the peritoneum; therefore, it is crucial to study disease initiation and progression using FTE-derived models. We previously demonstrated that loss of PTEN from the FTE leads to ovarian cancer. In the present study, loss of PTEN in FTE led to the enrichment of cancer stem cell markers such as LGR5, WNT4, ALDH1, CD44. Interestingly, loss of the transcription factor PAX2, which is a common and early alteration in HGSOC, played a pivotal role in the expression of cancer stem-like cells (CSC) markers and cell function. In addition, loss of PTEN led to the generation of two distinct subpopulations of cells with different CSC marker expression, tumorigenicity, and chemoresistance profiles. Taken together, these data suggest that loss of PTEN induces reprogramming of the FTE cells into a more stem-like phenotype due to loss of PAX2 and provides a model to study early events during the FTE-driven ovarian cancer tumor formation.

## Introduction

High-grade serous ovarian cancers (HGSOCs) are genotypically and phenotypically heterogeneous; however, they are almost all treated similarly with surgical debulking followed by paclitaxel and carboplatin (or cisplatin) chemotherapy. In addition, PARP inhibitors are used and have a high efficiency in treating homologous recombination-deficient ovarian cancer. The majority (70–90%) of HGSOC patients’ relapse, and ultimately succumb to the chemotherapy-resistant disease. Chemoresistant cells, present in the tumors, are hypothesized to be enriched for cancer stem cell-like properties, which may then contribute to cancer initiation and relapse^1^.

Several studies conducted on HGSOC have demonstrated that cancer stem-like cells (CSC) are characterized by a set of markers such as ALDH1, CD44, CD117(c-KIT), and CD133^2,3^. A large percentage of HGSOCs arise from fallopian tube epithelium (FTE) rather than the ovarian surface epithelium (OSE)^4,5^. Therefore, studying the CSCs from the FTE may improve our understanding of the pathways that lead to tumor formation and contribute to disease resistance.

*PTEN* is a tumor suppressor and its loss, either by deletion or reduced expression, contributes to ovarian cancer. PTEN is lost in 33% of early malignant lesions, known as serous tubal intraepithelial carcinomas (STIC)^6,7^, and is not detected in 30–50% of ovarian tumors^8,9^. PTEN expression is regulated by mutation, deletion, methylation^10^ and by microRNAs^11^ in ovarian cancer. Knockdown of *PTEN* alone by shRNA in murine oviductal epithelium (MOE; equivalent to human FTE) cells was sufficient to generate high-grade oviductal tumors with peritoneal colonization, in an allograft mouse model^12^. Furthermore, a floxed FTE-specific knockout of *Pten* deletion using the PAX8 promoter driving cre-recombinase was sufficient to induce ovarian cancer in a transgenic mouse model^13^. Even though the transgenic model did not progress to peritoneal metastasis, primary metastasis to the ovary was found in all mice, making this model relevant for studying early tumorigenesis^13^.

The WNT pathway has been linked with the development of chemoresistant and cancer stem cell-like populations of cells in the fallopian tube. For example, WNT4 was reported as a stem cell marker that was enriched in LGR5^+^ populations of the fallopian tube fimbriae^14^. *Pten* deletion from murine FTE cells resulted in upregulation of the WNT4 noncanonical pathway, which mediated FTE colonization of the ovary ex vivo^13^. DKK3 is an inhibitor of the WNT canonical pathway^15^, and its role in early tumorigenesis has not been addressed. DLL4 is expressed in ovarian cancer and it is shown to regulates tumor growth^16^. DKK3, ALDH1 and WNT4 are amplified in TGCA as compared to normal fallopian tube tissues (Supplementary Fig. 1). In addition to the WNT pathway, ALDH and CD44 are frequently considered as markers for CSCs in ovarian cancer cell lines^17,18^.

Modi et al. reported that cells with PTEN knockdown had reduced PAX2 levels and that re-expression of PAX2 reduced tumorigenesis in the PTEN-deficient model^19^. In the current study, we found that silencing PTEN in murine FTE increases CSC markers, which is partially dependent upon PAX2 loss. The pathways that generate these CSCs populations from the FTE have not been fully described. We found that loss of PTEN generated a heterogeneous cell population in terms of morphology and size, and based on size, these populations expressed different levels of CSCs markers and ability to form tumors. CSCs have previously been reported to display a peculiar increase in cell size, particularly in ovarian cancer^20,21^. This study gives new insight into the origins of FTE-derived ovarian cancer tumor cell heterogeneity due to loss of PTEN and PAX2, providing a model to study the mechanisms responsible for the emergence of cancer stem cells.

## Results

### Loss of PTEN in FTE causes enrichment in stem-like markers expression

**Fig. 1:**
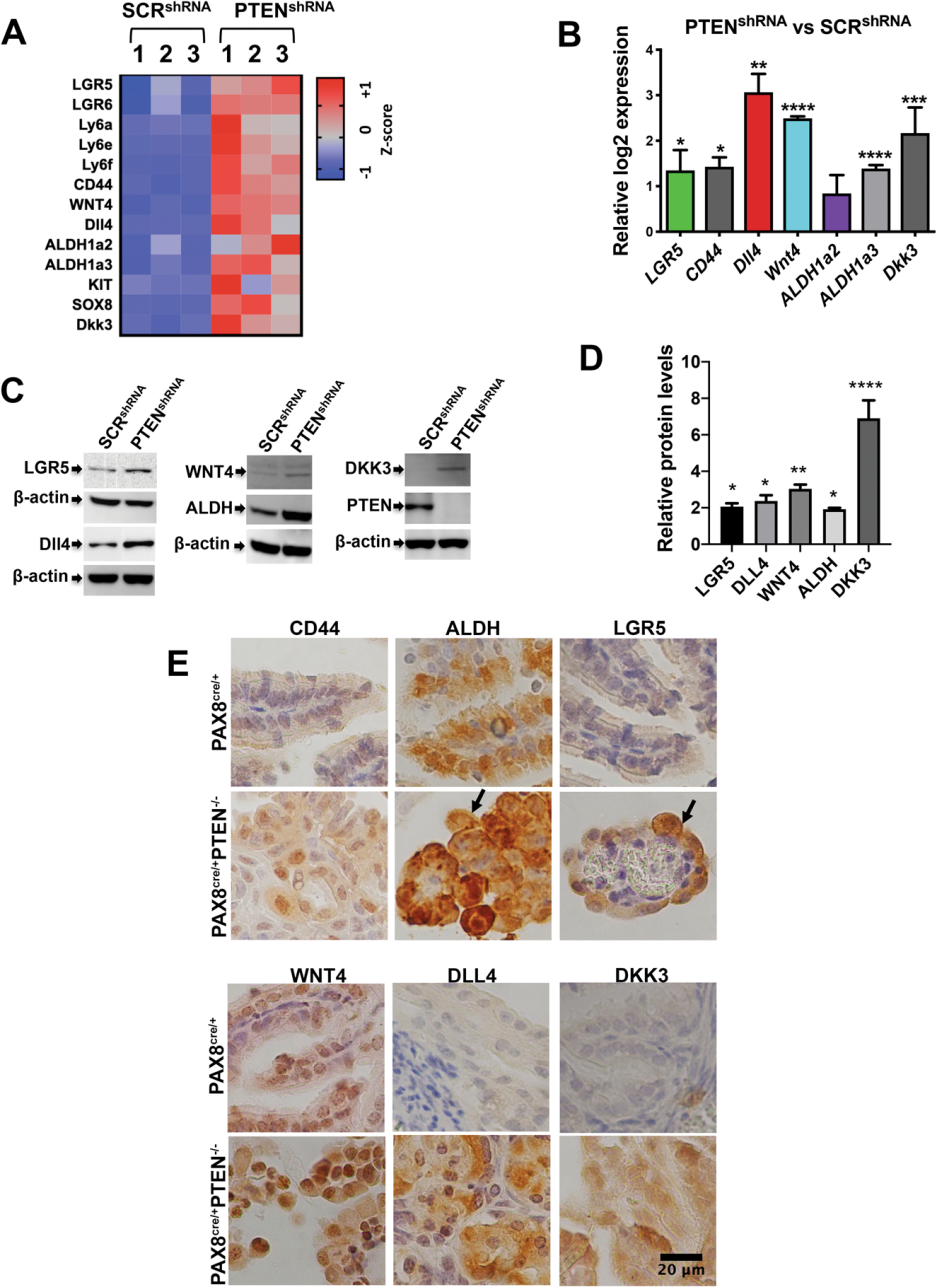
Loss of PTEN increases expression of CSC markers. **A** Heatmap from RNAseq data obtained from MOE cells depleted of *Pten* (named PTEN^shRNA^) compared to scrambled control (SCR^shRNA^). Only significantly regulated genes involved in CSC function are plotted (*p < 0.05). **B** qPCR validating mRNA levels in PTEN^shRNA^ compared to SCR^shRNA^. Three independent experiments, each repeated in three replicates were performed (*p < 0.05; ***p < 0.001). **C** Western blot of lysates from PTEN^shRNA^ and SCR^shRNA^ probed for DLL4, LGR5, PTEN, ALDH, and actin antibodies. **D** Western blot quantification of three independent experiments using ImageJ. Data were normalized to actin and fold changes over SCR^shRNA^ calculated. Statistical analysis performed using one-way ANOVA with multiple comparisons. **E** Immunohistochemistry (IHC) of tissues from control PAX8^cre/+^ and PTEN knockout *PAX8*^*cre/+*^*PTEN*^*fl/fl*^ transgenic mice were stained for DKK3, DLL4, ALDH, CD44, and LGR5 antibodies to determine protein expression. Scale bar represents 20 μm. Same observation were seen in three independent experiments using three different animals for PAX8^cre/+^ and three different animals *PAX8*^*cre/+*^*PTEN*^*fl/fl*^.

To uncover the mechanism of early tumorigenesis following reduction of *Pten* from murine oviductal cells (MOE), a previously performed RNAseq experiment was analyzed^13^ using MOE cells with stable expression of an shRNA targeting PTEN (MOE:*Pten*^shRNA^ that we will call PTEN^shRNA^). PTEN^shRNA^ monoclonal lines were isolated and used in this study. One clone with best reduction of PTEN was used, however other shRNAs targeting PTEN were previously tested in addition to the one used in this study to confirm they were responsible for similar phenotypes^13^. RNA sequencing of PTEN^shRNA^ revealed a panel of transcripts previously reported to be involved in cancer stem cell (CSC) function **(**Fig. 1A**)**. Two of these markers, LGR5 and WNT4, were reported as enriched in fimbriae stem cell populations^14^. In addition, ALDH1, a well-characterized marker associated with CSC in ovarian cancer^22,23^, was also increased. Several other CSC markers were altered in the RNAseq analysis included *Ly6a*, *Cd44*, *Cd117* (*c-kit*), and *Dll4* **(**Fig. 1A**)**. qPCR and western blotting validated the increase in a subset of these markers including CD44, ALDH1a3, LGR5 and WNT4 at the mRNA and protein level, respectively **(**Fig. 1B–D**)**. Flow cytometry of activated ALDH (AldeRed) showed an increase in PTEN^shRNA^ compared to SCR^shRNA^ **(**Supplementary Fig. 2B**)**. In addition, loss of *PTEN* in the human fallopian tube line, FT33, also increased ALDH protein levels **(**Supplementary Fig. 3C**)**. Protein levels of CD44, ALDH, LGR5, WNT4, DLL4, and DKK3 were elevated in tumors from *Pten* homozygous knockout mice (*Pax8*^cre/+^*Pten*^fl/fl^, transgenic model of ovarian cancer^13^) as compared to the normal fallopian (oviductal) tube epithelium **(**Fig. 1E**)**.

### Loss of PTEN in MOE:*Pten*^shRNA^ gives rise to clonogenic spheroids and retention of BrdU in vivo

**Fig. 2:**
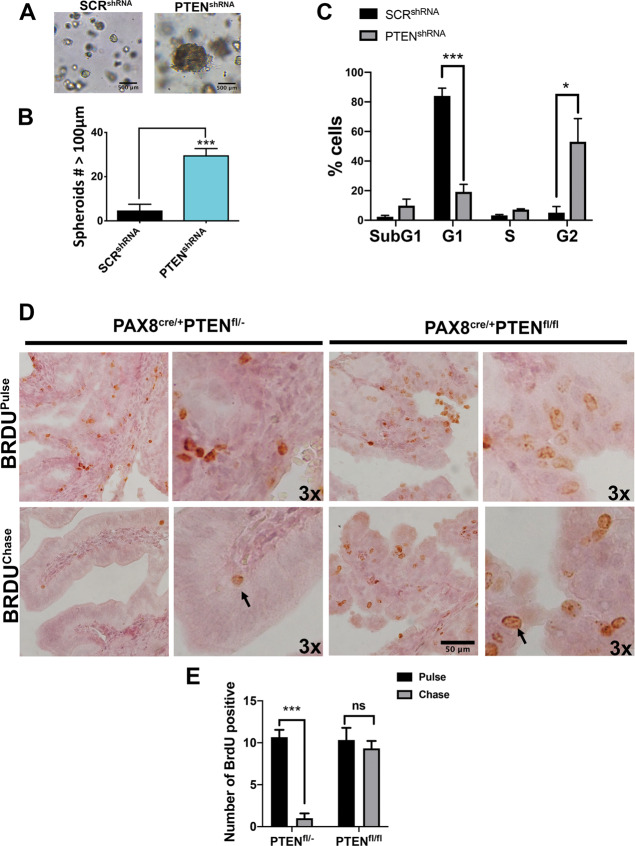
Loss of PTEN stimulates spheroid growth and enhances BrdU retention in a transgenic mouse model of fallopian tube-derived cancer. **A** MOE SCR^shRNA^ and PTEN^shRNA^ cells were grown in 50% matrigel and imaged using NIKON Eclipse TS100. **B** Number of spheroids with a diameter above 100 μm over the total amount of aggregates of three replicates in three independent experiments was quantified using Celigo (Nexcelom) (***p < 0.001). **C** SCR^shRNA^ or PTEN^shRNA^ were permeabilized and stained with PI for cell cycle analysis and analyzed using Cellometer K2 (Nexcelom). Statistical analysis was performed using Sidak’s multiple comparison in GraphPad. *p < 0.05; ***p < 0.001. **D** Mice were injected with BrdU and tissues were collected right after (pulse) injection or 2 weeks later (chase). Immunohistochemistry (IHC) using specific antibodies targeting BrdU were used in control *PAX8*^*cre/+*^ *PTEN*^*fl/+*^ and homozygous mice *PAX8*^*cre/+*^*PTEN*^*fl/fl*^. Scale bar represents 50 μm. The 3× label indicate a further magnification to show difference in cell size; arrows indicate larger cells. **E** Number of cells with positive BrdU staining were counted in three different fields for three independent experiments. Two-way ANOVA with multiple comparisons was applied. *p*-value*** = 0.0003.

To determine whether the increase in CSC markers also increased CSC function, a multispheroid matrigel assay was performed using MOE:*Pten*^shRNA^ cells^12,13^. Loss of PTEN increased spheroid formation in matrigel compared to the scrambled control (SCR^shRNA^) **(**Fig. 2A, B**)**. To test whether loss of PTEN-induced clonogenic spheroids, *Pten*^shRNA-GFP^ MOE cells labeled with GFP, and *Pten*^shRNA-RFP^ cells labeled with RFP, were co-cultured in matrigel. All spheroids that grew in the matrigel assay were either entirely GFP or entirely RFP based on fluorescence microscopy **(**Supplementary Fig. 2A**)** suggesting that the spheroids were derived clonally. Cell cycle analysis found that shRNA directed against PTEN caused an arrest in the G2 phase **(**Fig. 2C**)**. In addition, a BrdU-label retaining pulse-chase assay was performed in vivo in the benign heterozygous *Pax8*^*cre/+*^*PTEN*^*fl/+*^ and the tumorigenic homozygous *Pax8*^*cre/+*^*Pten*^*fl/fl*^ oviducts. IHC of the nuclear BrdU staining demonstrated a significant higher number of the dye retaining cells in the homozygous *Pten* deleted oviducts, suggesting the presence of stem-like populations that are typically long dye retaining cells **(**Fig. 2D, E**)**.

### Morphologically distinct subpopulations of MOE:*Pten*^shRNA^ cells present a different CSC profile and chemoresistance

**Fig. 3:**
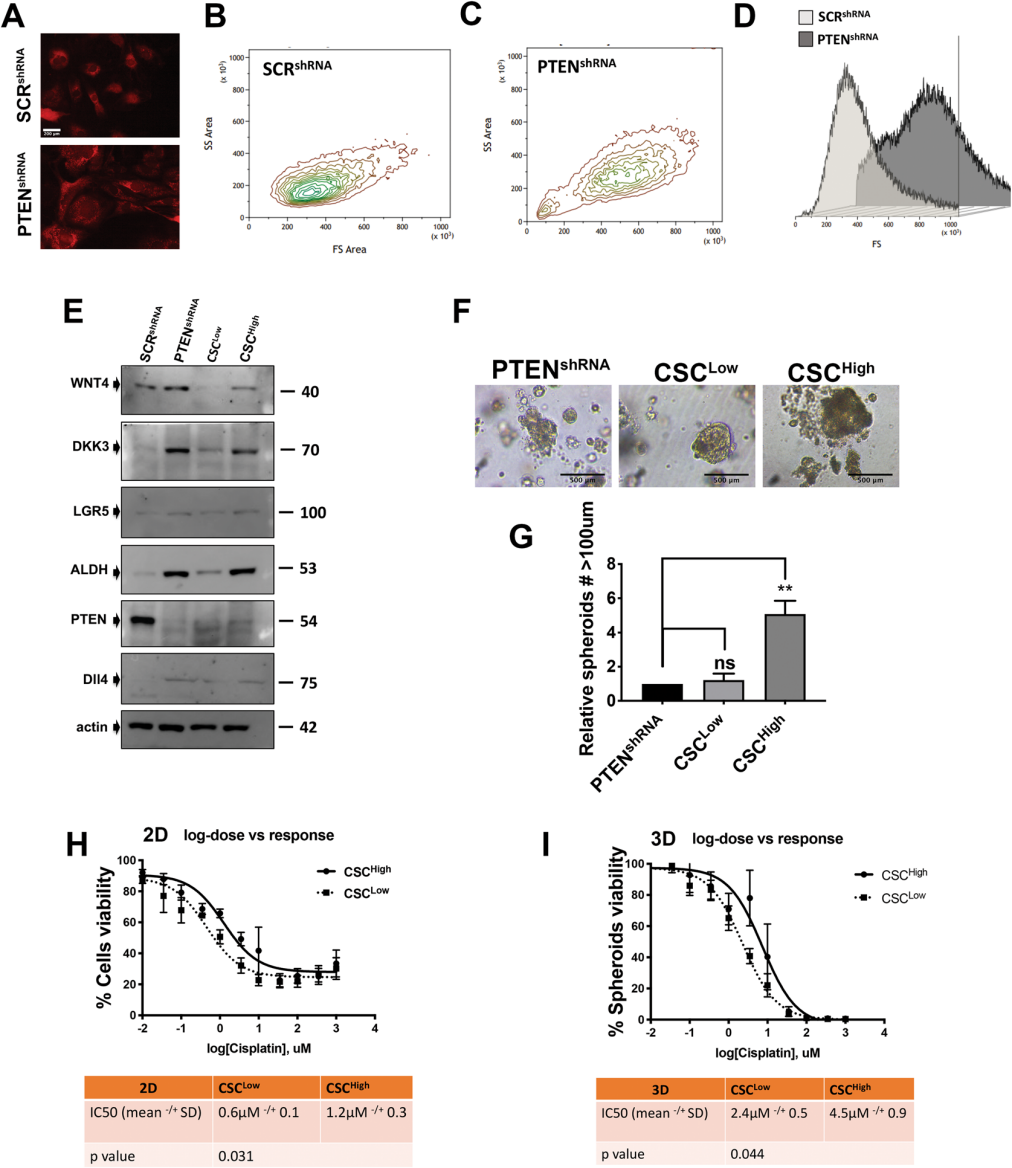
Loss of PTEN generates two distinct populations of cells with differential CSC properties. **A** Representative image of SCR^shRNA^ and PTEN^shRNA^ cells expressing RFP were taken with fluorescence microscope showing larger cell size upon PTEN depletion. **B** Flow cytometry image showing gating of SCR^shRNA^ cells in side scatter (SSC) vs. forward scatter (FSC) channels to show relative difference in size. In green is the population with highest density and correspondent size. **C** Flow cytometry image showing gating of PTEN^shRNA^ cells in (SSC) vs. (FSC) scatter channels to show relative difference in size in FS. In green is the population with highest density and correspondent size in FS. **D** Flowcytometry generated overlapping graphs to show the difference in size between SCR^shRNA^ and PTEN^shRNA^. **E** Western blot of sorted PTEN^shRNA^ cell populations based on size. Large cells are named CSC^High^ and small cells are named CSC^Low^ side by side with SCR^shRNA^. Specific antibodies were used to probe for CSC markers including ALDH, WNT4, LGR5, DKK3, PTEN, DLL4, and actin. **F**, **G** PTEN^shRNA^, CSC^High^, and CSC^Low^ were grown in 50% matrigel and imaged. Scale bar represents 500 μm. Quantification of number of spheroids with diameter above 100 μm was performed using Celigo (Nexcelom) (**p < 0.01). Fold changes over PTEN^shRNA^ were calculated. **H** CSC^High^ and CSC^Low^ were grown as a monolayer and treated with different concentrations of cisplatin for 72 h and MTT assay was performed. Three independent experiments were averaged for the IC_50_. *p* < 0.05. **I** CSC^High^ and CSC^How^ were grown as spheroids in ultralow attachment with round bottom wells and treated with different concentrations of cisplatin for 10 days and Promega 3D viability assay was performed. Three independent experiments were averaged for the IC_50_. *p < 0.05 (as shown in the tables below).

The *PAX8*^*cre/+*^*PTEN*^*fl/fl*^ mouse model presented enlarged nuclei **(**Figs. 1E–2D arrows**)**. Enlarged cells have been associated with CSC function in ovarian cancer^24^. We found that loss of PTEN in the MOE:*Pten*^shRNA^ cells induces a heterogeneous population of cells. Some cells in this population were larger than the wild-type control cells expressing PTEN **(**Fig. 3A–D**)**. PTEN^shRNA^ presented two live populations of cells with distinct size. The highest density of the population was between 400 and 600 (as represented by the green color) whereas the SCR^shRNA^ presented only one population between 200 and 400 **(**Fig. 3B, C). Enlarged cells in human ovarian cancer, suggested to be enriched in cancer stem cell markers^24,25^, have been proposed to be predictors of poor prognosis^21^. We hypothesized that the enlarged subpopulation in our PTEN-deficient model could have increased CSC potential and that size could be a tool to isolate and study CSCs. To test this possibility, two subpopulations of cells with different sizes were isolated from MOE:*Pten*^shRNA^ cells using flow cytometry-based cell sorting **(**Fig. 3C**)**. All cells small and big were alive, they were replaced in culture, and displayed the same growth rate **(**Supplementary Fig. 2C**)**. The expression of CSC markers was then determined using Western blotting, and it was found that the population of enlarged cells expressed more abundant levels of the CSC markers **(**Fig. 3E**)** and presented with a cell cycle profile similar to the PTEN^shRNA^, specifically, reduced cells in G1 and increased cells in G2 compared to the population with smaller cells **(**Supplementary Fig. 2D**)**. Therefore, we named the population with smaller cells, CSC^Low^ and the population with enlarged cells we called CSC^High^ to denote the populations that express lower and higher CSC markers. CSC^Low^ and CSC^High^ will stand for PTENshRNA^CSCLow^ and PTENshRNA^CSCHigh^, so they are always in the PTENshRNA background. Additionally, even though SCR^shRNA^, which is a nontumorigenic line, have a more homogeneous cell size, we isolated smaller and bigger cells from it, and showed that the bigger cells, do not present enrichment in CSC **(**Supplementary Fig. 3F**)**. Therefore, we focused on tumor-forming PTEN^shRNA^ cells and isolated the small and the enlarged populations by flow cytometry. To assess CSC function we performed a spheroid assay in matrigel and demonstrated that the CSC^High^ population had an increased ability to form spheroids compared to the mixed PTEN^shRNA^ population and CSC^Low^ **(**Fig. 3F, G**)**. CSCs have also been suggested to be mediators of chemoresistance and tumor progression. To test chemoresistance, CSC^Low^ and CSC^High^ cells were plated as a 2D monolayer or as 3D spheroids and were exposed to a range of cisplatin concentrations. Consistent with the literature, the CSC^High^ population was more resistant to cisplatin in both 2D and 3D assay **(**Fig. 3H, I**)**.

### The CSC^High^ population had higher tumorigenic potential in vivo and increased ability to attach to the ovarian stroma

**Fig. 4:**
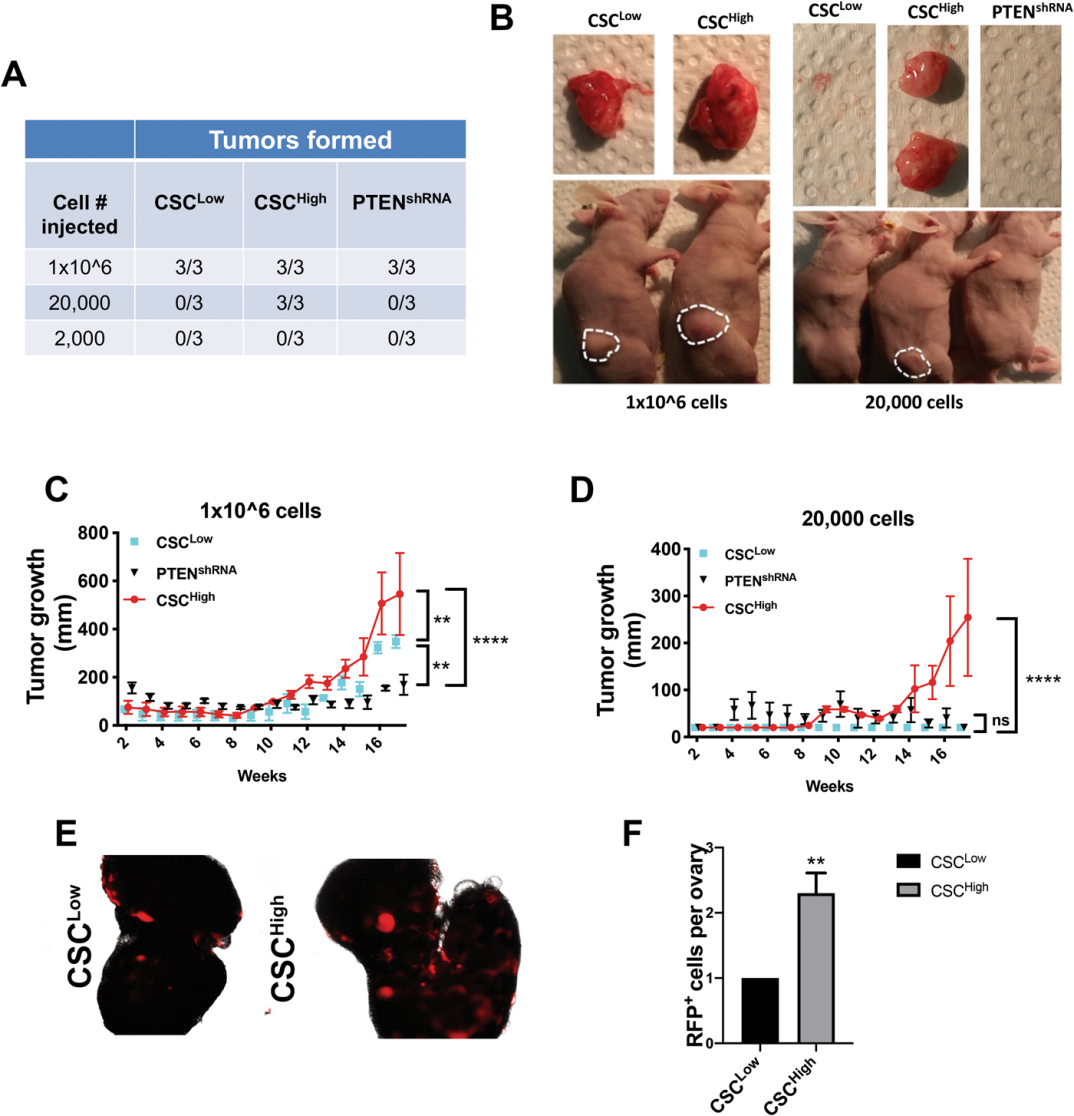
CSC^High^ demonstrates increased tumorigenic capabilities in vivo and increased adhesion to the ovary. **A** Table showing the number of mice forming tumors in the different concentrations’ groups injected with PTEN^shRNA^, CSC^High^, or CSC^Low^. **B** Images comparing tumors from CSC^Low^ and CSC^High^ at 1 × 10^6^ of cells and 20,000 cells injected. **C**, **D** Tumor growth over time was measured by calipers for 1 × 10^6^ cells and 20,000 cells injected. Statistical analysis was performed using two-way ANOVA. **p < 0.01; ****p < 0.0001, ns nonsignificant. **E**, **F** Wounded ovaries incubated with PTEN^shRNA^-RFP cells for 24 h. Cells that attached to the ovary were counted using a NIKON Eclipse TS100. Three replicates in three independent experiments were quantified using Celigo (Nexcelom). Statistical analysis was performed using unpaired *t*-test. **p < 0.01.

CSCs have been reported to form tumors at cell concentrations 100 times less than control cells. To test whether the CSC^High^ cells were more tumorigenic, CSC^Low^, CSC^High^ and the PTEN^shRNA^ mixed population were injected subcutaneously into nude mice at a density of 2000, 20,000, and 1 × 10^6^ cells/mouse **(**Fig. 4A**)**. All mice developed tumors when grafted with 1 × 10^6^ cells **(**Fig. 4A–C**)**; whereas only the CSC^High^ cells formed tumors at 20,000 cells **(**Fig. 4B–D**)** suggesting that this population is more tumorigenic. In addition, there was significant increase in tumor size when CSC^High^ were cell injected **(**Fig. 4C, D**)**. Injection of 2000 cells did not produce any tumors by six months. The ability of CSC^Low^ and CSC^High^ cells to attach to the ovary was evaluated by performing an ex vivo ovary adhesion assay using RFP labeled CSC^Low^ and CSC^High^ MOE cells. The CSC^High^ population had enhanced adhesion based on cell number to a wounded ovary, exposing the stroma, as compared to the CSC^Low^ suggesting that the CSC^High^ are more efficient at colonizing the ovary **(**Fig. 4E, F**)**.

### Acquisition of a CSC-like profile was dependent on mTOR activation

**Fig. 5:**
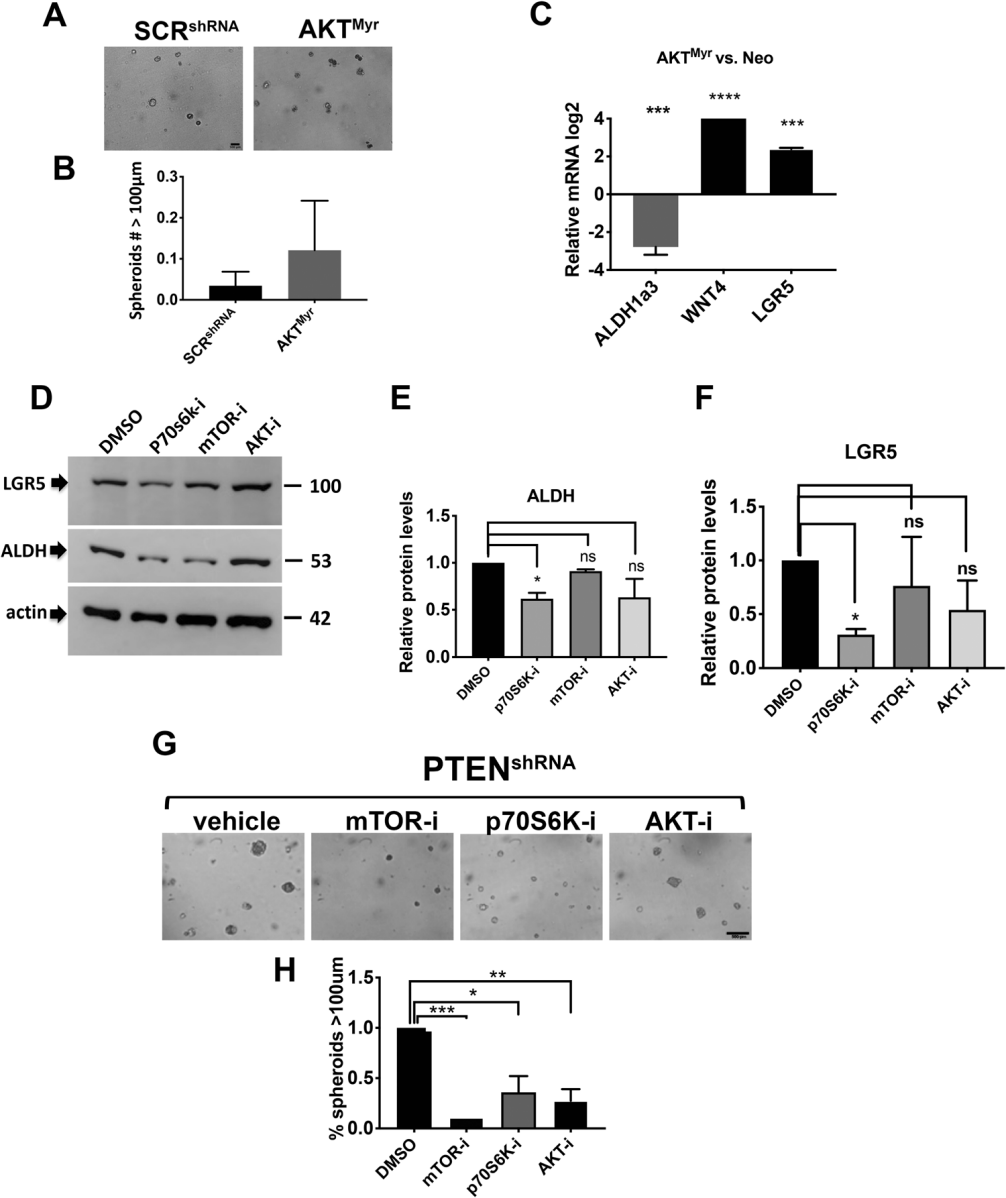
mTOR activation is critical for CSC function. **A**, **B** Spheroid formation in matrigel of control cells and constitutively active AKT^Myr^ were imaged. Scale bar represents 500 μm. Number of spheroids with diameter higher than 100μm were counted using Celigo (Nexcelom). Statistical analysis was performed using one-way ANOVA. **C** Control cells and constitutively active AKT^Myr^ were seeded, RNA was extracted, and qPCR performed in three independent experiments ***p < 0.001; ****p < 0.0001. **D–F** PTEN^shRNA^ cells were treated with DMSO as a vehicle or pAKT inhibitor, MK2206 (10 μM), pmTOR inhibitor, Rapamycin (20 nM), or pP70S6k inhibitor, PF-04708671 (25 μM) and processed for Western blots to detect ALDH and LGR5. Quantification of western blots from three independent experiments was performed using ImageJ and normalized by actin. *p < 0.05. **G**, **H** Spheroid formation in matrigel of PTEN^shRNA^ cells treated with DMSO vehicle or pAKT inhibitor, MK2206 (10 μM), pmTOR inhibitor, Rapamycin (20 nM), or pP70S6K inhibitor, PF-04708671 (25 μM). Scale bar represents 500 μm. Spheroids were imaged and the number with a diameter higher than 100μm were counted using Celigo (Nexcelom) *p < 0.05, **p < 0.01, ***p < 0.001.

Since loss of PTEN results in AKT activation^12^, we investigated whether the increase in CSC markers in the MOE*:Pten*^*s*hRNA^ was mediated by activated AKT. We employed MOE cells stably transfected with a constitutively activated myristoylated AKT construct (AKT^Myr^) and found that this model did not result in spheroid formation in matrigel **(**Fig. 5A, B**)** and did not have increased ALDH mRNA expression **(**Fig. 5C**)**. These data suggest that activation of AKT alone may not be sufficient to induce CSC characteristics. However, activation of AKT did increase LGR5 and WNT4 expression indicating that it may support increased expression of some CSC markers independent of spheroid formation **(**Fig. 5C**)**. Previous studies have shown that AKT^Myr^ was also unable to generate multicellular tumor spheroids in ultralow adhesion plates^26^ or tumors in allograft models^12^.

To address the contribution of AKT and mTOR1 on CSC function, MOE:PTEN^shRNA^ cells were treated with inhibitors targeting AKT (MK2206), mTOR (Rapamycin) or P70S6K, downstream of mTOR1 (PF-04708671) and CSC markers expression was evaluated. The AKT inhibitor (MK2206) did not reduce CSC marker expression. Inhibition of P70S6K significantly inhibited both LGR5 and ALDH1a3 expression **(**Fig. 5D–F**)**. Treatment of PTEN^shRNA^ cells with the AKT inhibitor, mTOR inhibitor, and P70S6K inhibitor decreased spheroid formation **(**Fig. 5G**)**. Only inhibition of P70S6K significantly inhibited spheroids formation and reduced expression of ALDH and LGR5.

### CSCs enrichment from PTEN loss is partially mediated by loss of PAX2

**Fig. 6:**
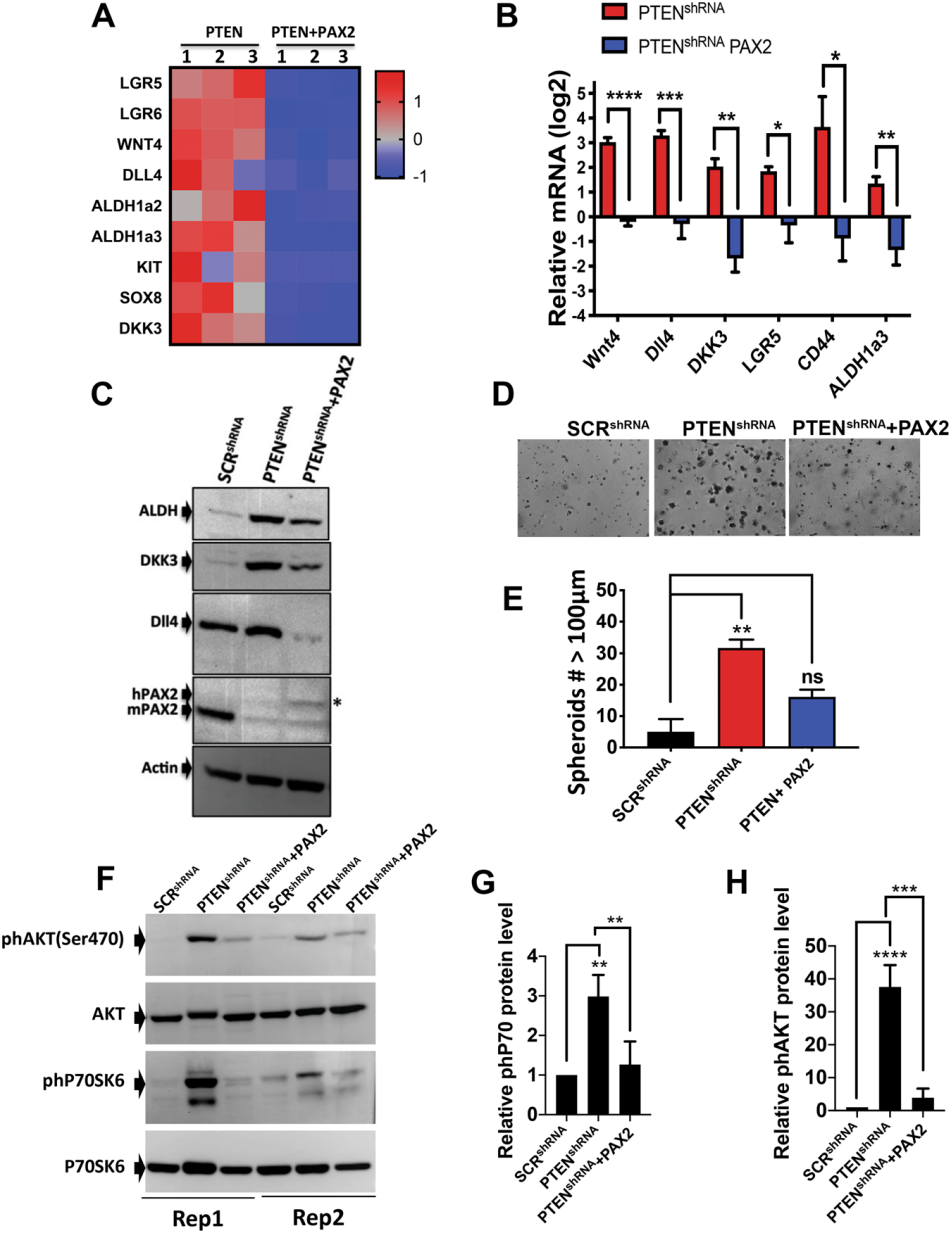
Re-expression of PAX2 reverses the increase in cancer stem cells markers induced by loss of PTEN. **A** Heatmap with genes regulated in PTEN^shRNA^ vs. PTEN^shRNA^ plus PAX2, showing that re-expression of PAX2 decreases CSC markers. **B** qPCR validating that re-expression of PAX2 decreases CSC markers in three independent experiments. Statistical analysis was performed using two-way ANOVA; *p < 0.05, **p < 0.01, ***p < 0.001, ****p < 0.0001. **C** Western blot comparing CSC protein levels in SCR^shRNA^, PTEN^shRNA^, and PTEN^shRNA^ plus PAX2 using antibodies against ALDH, DKK3, DLL4, and actin. **D**, **E** Spheroid formation in matrigel imaged with a Nikon Eclipse TS100. Number of spheroids with diameter higher than 100 μm were counted using Celigo (Nexcelom) **p < 0.01. **F–H** SCR^shRNA^, PTEN^shRNA^, and PTEN^shRNA^ + PAX2^shRNA^ cells were subjected to western blot for phosphorylated P70S6K or AKT and three independent experiments quantified using ImageJ. Two out of the three biological replicates experiments are showed as Rep1 and Rep2. Normalization by actin was performed and statistical analysis using one-was ANOVA; **p < 0.01, ***p < 0.001, ****p < 0.0001.

Loss of PAX2 is the one of the earliest molecular events detected in HGSOC progression from normal FTE^27^. We performed a novel RNAseq analysis comparing PTEN^shRNA^ and PTEN^shRNA^ re-expressing PAX2 (PTEN^shRNA^ + PAX2). The analysis revealed that PAX2 re-expression blocked loss of Pten-induced CSC markers expression **(**Fig. 6A**)**. To validate if re-expression of human PAX2 in PTEN^shRNA^ cells could inhibit CSCs marker expression, the transcripts from the RNAseq were validated by qPCR and western blots. Re-expression of PAX2 reduced CSCs markers at the mRNA and protein level **(**Fig. 6B, C**)**. These data are consistent with the literature where loss of PAX2 increased the CSC marker CD44^28^ and increased ALDH^29^. Overexpression of PAX2 in SCR^shRNA^, which already express endogenous PAX2, did not alter mRNA expression of the CSC markers **(**Supplementary Fig. 3E**)**. These data suggest that loss of PAX2, which occurs when PTEN is silenced^19^, contributes to CSCs enrichment. PAX2 re-expression also significantly reduced spheroid formation **(**Fig. 6D, E**)** and reduced loss of PTEN-induced increase in cell size **(**Supplementary Fig. 3D**)**. Re-expression of PAX2 reduced phosphorylation of P70S6K and AKT **(**Fig. 6F–H**)**, suggesting that downregulation of PAX2 might mediate CSC function and survival through those pathways. To address direct involvement of PAX2 in CSC regulation, we measured the expression of CSC markers upon PAX2 knockdown by shRNA in MOE cells. We found that PAX2 knockdown by shRNA increased CSC markers at the mRNA and protein level suggesting that loss of PAX2 plays a role in cancer stem cell function **(**Supplementary Fig. 3A, B**)**.

## Discussion

In this study, loss of PTEN in FTE cells induced an increase in CSC markers and spheroid formation compared to scramble control. We also found that loss of PTEN in FTE cells resulted in subpopulations of cells with distinct morphology and used cell size as tool to isolate CSC. We demonstrated that cells with reduced PTEN and increased size had increased CSC markers, were more chemoresistant, and able to form tumors in vivo when inoculated at lower cell concentration. In a previous study, loss of PTEN by shRNA in FTE cells is sufficient to induce tumorigenesis compared to a scramble shRNA control unable to form tumors^12,13^. Murine FTE cells with reduced PTEN showed lower PAX2 mRNA and protein levels compared to the scrambled control^19^. We found the re-expression of PAX2 could reverse many of the CSC features including the expression of the CSC markers as well as the formation of spheroids. PAX2 knockdown in normal fallopian tube epithelial cells expressing PTEN also increased CSC markers such as ALDH, DKK3, and DLL4 confirming the critical role of PAX2 reduction on CSC function. The reversal of these CSC features from PAX2 re-expression coincided with the inhibition of phosphoP70SK6 and phosphoAKT.

Loss of PTEN has been reported to impact stem cell self-renewal^30,31^. LGR5 and WNT4 have been reported to be co-enriched in stem cell-like populations in oviducts^14^. ALDH and CD44 have also been extensively characterized as CSC markers in HGSOC^17,18^. The current study provided a link to how these markers may change in that ALDH expression was increased upon silencing of PTEN, which is consistent with stem cell-like capacity. Importantly, from a translational perspective, Buckanovich et al. reported that ALDH inhibitors were able to induce cell death in ovarian cancer stem cells and reduce tumor cell survival^32^ suggesting that not only are these markers important for characterizing the cancer stem cell population; but also blocking their expression has an impact on their tumor-forming capabilities. Ng et al.^14^ do not confirm increase in ALDH1a1 in the potential fallopian tube stem cell niche but a modest increase in ALDH1a2; ALDH1a3 however, was not assessed. Lack of increased ALDH1a1 in early tumorigenesis is supported also by Chui H.M. et al.^33^. Interestingly, in the Chiu paper, they analyzed fallopian tubes with early lesions (BRCA mutation and consequent p53 stabilization) and did not find increase in ALDH1a1. However, we know that BRCA mutation and p53 stabilization are not sufficient to generate ovarian cancer, but rather they are precursors. It is the addition of other pathway alterations that drive tumorigenesis, such as loss of PTEN or regulation of the pathway downstream of PTEN. In addition, the role of all ALDH1 isoforms have not been addressed and here we found that ALDH1a3, not ALDH1a1 has been upregulated. Our findings suggest that ALDH1 upregulation could be acquired following downregulation of PTEN. In our case, loss of PTEN generated two populations (at least) of cells with different CSC profiles, both tumorigenic with the enlarged cells having increased tumorigenicity and chemoresistance. Therefore, loss of PTEN leads to tumorigenic cells with a subpopulation of enriched cells, consistent with heterogeneity in human tumors.

DKK3’s role in CSC has not been reported and its role in cancer has been controversial^15,34^. DKK3 is an inhibitor of the WNT canonical pathway^15^. We previously showed that the WNT4 and the noncanonical WNT pathway is activated in MOE:PTEN^shRNA^ and that it contributes to cell motility and ovarian adhesion, which is thought to occur when fallopian-tube-derived tumor cells initially colonize the ovary^15^. One study reported that DKK3 is reduced in HGSOC as compared to fallopian tube control^34^. However, an analysis of the RNA expression of the Singapore dataset for HGSOC patients revealed upregulation of DKK3 when HGSOC is compared to fallopian tube **(**Supplementary Fig. 1**)**.

The majority of tumors present with cellular heterogeneity including a population of large cancer cells, increased DNA copy number, and resistance to chemotherapy^35^. In ovarian cancer, these giant cells are predictors of poor prognosis^21^ and have been associated with CSCs^24,25^. Despite all the cell populations having reduced PTEN expression, the MOE:PTEN^shRNA^ cells consistently had two sizes of cells. Loss or mutation of *PTEN* was previously associated with increased cell size through a PI3K-dependent and independent pathway^36^. Loss of PTEN has been associated with cell size arrest induced by DNA-damage^37,38^ and spindle orientation through PLK1, resulting in increased size and DNA copy number suggesting that loss of PTEN may generate CSCs through asymmetric division^39^. Mutation of PTEN causes hamartoma syndrome, where patients display an increased incidence of cancer and enlarged cells^40^. The stem-like subpopulation represents a potential target to be tested for therapeutic purpose.

The most frequent mutation in HGSOC occurs in *TP53* and appears in precursor lesions^41^ and the loss of *PAX2* is also reported to be lost in early fallopian tube tumorigenesis^27^. PAX2 is critical for FTE differentiation^42^ and thus its loss lead to less differentiated, more stem-like cells^1^. However, PAX2 is not methylated or mutated in HGSOC^43^, but rather transcriptional regulated. Wild-type TP53 has been shown to activate PAX2 transcription whereas the R273H TP53 mutation has been proven to suppress it^19^. Loss of *PAX2* or mutation of *TP53* in the oviduct alone is not sufficient to induce tumorigenesis^19^ and precursor lesions frequently present also reduced or mutated PTEN expression^6,19^. Taken together, the loss of PTEN in FTE leads to stabilization of p53 and loss of PAX2^12,13,39^. Interestingly, most mouse models of FTE-derived ovarian cancer require loss of PTEN to induce tumor formation, and in all of them, the loss of PTEN causes tumors to form earlier^44,45^. In addition, re-expression of PAX2 in PTEN-depleted FTE cells blunted tumor formation, suggesting that loss of PAX2 is important in PTEN-induced tumorigenesis^19^. PAX2 has been shown to inhibit the CSC marker CD44 in MOE cells^3^. We demonstrated that re-expression of PAX2 reduces CSCs enrichment from loss of PTEN based on spheroid formation and CSC markers expression. The repression of CSC properties may be part of the reason that PTEN^shRNA^ cells re-expressing PAX2 are less aggressive at forming tumors in vivo as compared to PTENshRNA models^19^.

Furthermore, our study suggests that both mTOR and AKT activation, which are activated when PTEN is silenced, play a role in CSC marker expression and spheroid formation. Our data also show that inhibitors of AKT reduce spheroids formation; however, a constitutive active myristoylated AKT that is confined to the plasma membrane does not inhibit CSC function. This suggests a role for nuclear AKT in CSC function, consistent with findings in breast cancer^46^. Direct inhibition of p70S6K and AKT, that are frequently activated in HGSOC^47,48^ with small molecule inhibitors might be a reasonable therapeutic strategy to impact stemness in addition to PAX2 re-expression^49,50^. Previous studies screened for several compounds able to increase PAX2 expression, and found potential hits, such as luteolin^19^.

In conclusion, the evidence reported here, suggests a role for loss of PTEN in increasing CSC properties via an enrichment of CSC markers confined in an enlarged population of cells with increased chemoresistance. In addition, our study supports a role for PAX2 re-expression in HGSOC as a mechanism to reduce chemoresistant cancer stem-like cells function and tumor relapse.

## Material and methods

### Cell culture

MOE were originally provided by Dr. Barbara Vanderhyden, U. of Ottawa, and they were not immortalized using hTERT or SV40 or any transgenes but spontaneously. Murine cells have the ability to grow on plastic for several passages, which along with culture in estradiol and EGF allows for the culture of these cell lines. FT33 were provided by Ronny Drapkin^51,52^, and are immortalized with hTERT and SV40. FT33 cells were maintained, as previously described^53,54^ in grown in DMEM-Ham’s F12 supplemented with penicillin/streptomycin and 2% Ultroser-G (Crescent Chemicals). MOE cells were grown in the DMEM with 10% FBS (Gibco), L-glutamine (2 mmol/L, Gibco), EGF (0.1 mg/mL, Roche), ITS (Roche), gentamicin (50 mg/mL, Gibco), B-estradiol (1 mg/mL in 100% EtOH, Sigma–Aldrich), and penicillin/streptomycin.

FT33 were transfected using TransIT LT1^TM^ as described previously^12^ with plasmids containing the shRNA targeting PTEN and the control nontargeting shRNA. MOE were previously generated^12,13^ (shRNA used were: TRCN0000322421 and SHC002 for murine cells and TRCN0000002749 and SHC016-1EA for human cells from Sigma). Single clones were isolated from FT33 control and PTENshRNA as well as from MOE PTENshRNA and control shRNA transfected MOE cells (as previously described^12,13^). All of the cells were passaged a maximum of 20 times and cultured in the monolayers in 95% air and 5% CO_2_ at 37 °C cell incubator (Sanyo, Japan) according to the ATCC cell culture protocol. The shRNA lines media contained puromycin for maintenance.

Rapamycin was obtained from Sigma and used at 20 nM final; P70S6K inhibitor (PF-04708671) and Akt inhibitor (MK2206) were obtained from Cayman and used at 25 and 10 μM final, respectively.

### Animal care

All animals were treated in accordance with the National Institutes of Health and institutional (UIC) Guidelines for the Care and Use of Laboratory Animals. Mice were housed under normal condition environment and provided food and water ad libitum. The C57b/6 *LoxP-PTEN-LoxP* obtained from MMHCC (Mouse Models of Human Cancer Consortium) were bred with mice expressing cre-recombinase under control of PAX8 promoter from Research Institute of Molecular of Pathology, Vienna, Dr. Bohr-Gasse^55^.

### BrdU pulse-chase assay

For pulse-chase BrdU experiments, six mice *Pax8*^cre/+^*Pten*^fl/+^(heterozygous) or *Pax*8^cre/+^*Pten*^fl/fl^(homozygous) were injected with 100 mg/Kg BrdU every day for 1 week. The age of the transgenic mice for injections was 6 weeks. A set of mice (#3) was sacrificed right after injection and another set was kept without further injections, so that BrdU could be chased out of the cells for 2 weeks. The reproductive tissues were isolated, fixed in 4% PFA and processed for IHC. Antibody against BrdU (Abcam) was used with overnight incubation. Cells with positive staining BrdU per field were counted. Three fields from three independent experiments were counted and plotted using prism.

### Limiting dilution assay

Subcutaneous injections (2000, 20,000, and 1 × 10^6^ cell) were done in nude mice. The age of the mice for subcutaneous injections was 6 weeks. With 95% confidence level and 50% confidence interval (considering a difference between groups of 200 and a sigma 50 for expected standard deviation) on a population of 27 mice the number of mice required for a power >80% was 3. Therefore, a number of nine mice per cell line were used with three mice per cell dilution. Tumor size was measured by caliper every week for 6 months. The mice were all female for the purpose of studying ovarian cancer.

### RNA isolation, cDNA synthesis, and RT-PCR

RNA extraction was performed as previously described^56^. iScript^TM^ cDNA synthesis kit (BioRad, Hercules, CA) and SYBR green (Roche, Madison, WI) were used according to manufacturer’s instructions. All qPCR runs were performed on the CFX96 (Biorad, San Francisco, CA). Primers used are described in Table SI. For RNAseq experiments RNA was isolated using RNAeasy kit from Qiagen and submitted to the Northwestern NUseq Core and submitted to GEO: GSE157358.

### Western blot analyses

For the study with inhibitors, the cells were seeded at 1 × 10^6^/10 cm dish and treated the day after with 20 nM Rapamycin, 10 μM MK2206, 25 nM PF-04708671, or DMSO for 72 h. Cells were lysed in RIPA buffer (50 mM Tris pH 7.6, 150 mM NaCl, 1% Triton X-100, 0.1% SDS) with protease (Sigma) and phosphatase inhibitors (tablets from Roche). Protein lysates (30 μg) were loaded onto a SDS-PAGE and transferred to nitrocellulose membrane. Blots were blocked with 5% milk or BSA in TBS-T and probed at 4 °C overnight with primary antibodies (Table SII). Membranes were incubated with anti-rabbit or anti-mouse HRP-linked secondary antibody for 30 min and developed as described previously^56^.

### Immunohistochemistry (IHC)

Tissues were fixed in 4% PFA, embedded in paraffin, processed and prepared for immunohistochemistry as previously reported^12,53,57^. The age of the transgenic mice for IHC was 4 months. Primary antibodies were incubated overnight (Table SII). Images were acquired on a Nikon Eclipse E600 microscope using a DS-Ri1 digital camera and NIS Elements software.

### Flow cytometry and cell sorting

Cells were seeded in 10 cm dishes and incubated at 37 °C until 90% confluent. Then the cells were washed with PBS and trypsinized. Cells were collected in complete medium and counted 1 × 10^7^ cells were washed in PBS and resuspended in in 500 μl of Basic Sorting Buffer (Phosphate Buffered Saline (Ca/Mg^++^ free)), 1 mM EDTA, 25 mM HEPES pH 7.0, 1% Fetal Bovine Serum (Heat-Inactivated), 0.2 μm filter sterilize) and kept on ice until analysis. Cells were sorted using Astrio and collected in Collection Medium containing 25% FBS.

### ALDH activity

MOE cells SCR^shRNA^ or PTEN^shRNA^ were plated at 3 × 10^5^/ml density. All reagents were prepared as reported in the manufacturer instructions. The day after plating, cells were treated with AldeRed substrate (AldeRed 588-A, Sigma) or with ALDH inhibitor DEAB. After 1-h incubation at 37 °C, cells were trypsinized, centrifuged, washed and imaged using flow cytometry. Gates were adjusted in cells treated with AdeRed substrate to eliminate background and to setup baseline.

### Cell size analysis

MOE cells SCR^shRNA^, PTEN^shRNA^, or PTEN^shRNA^ + PAX2 were plated at 1 × 10^6^/ml. The day after the cells were trypsinized, centrifuged and washed in PBS. Twenty microliter of cell suspension was added onto the Cellometer K2 (Nexcelom) slides and cell count and diameter per each cell was measured by the machine. Data were presented in prism as percent per diameter.

### Matrigel multispheroid assay

Cells were seeded at 1000 per well in 50% Matrigel and 50% complete medium for MOE cells^12^. To avoid the cells to attach to the bottom of the plate, 96-well ultralow flat bottom attachment plates were used (Corning). Images were taken from three wells (technical replicates) and experiments were repeated independently three times. The cells were incubated at 37 °C for 12 days and analyzed using Celigo (Nexcelom). The protocol we used, called colony (1 + 2 + 3 + 4 + 5, merge) allowed z-stack through the agarose and stitching of the images to create a final picture that included combination of all spheroids formed. The machine gave measurements of number of spheroids and the area of single spheroids from which we calculated the diameter.

### Cell cycle assay

Cells were seeded at 1 × 10^6^ in 10 cm dish. When cells reached 90% confluency, they were trypsinized, washed with PBS, fixed with Ethanol 70% for 1 h, washed in PBS, and then treated with RNAase (100 μg/ml) for 15 min at room temperature (RT). Propidium iodide (100 μg/ml) was then added and incubated at RT for 45 min, after which cells were washed with PBS and finally resuspended in 200 μl of PBS. Cell cycle was analyzed using Cellometer (Nexcelom) that generate an histogram per each sample in the FCS software. Gating was manually optimized and percent of cells in each phase were then plotted using prism.

### Viability assay

For adherent conditions, cells were seeded at 2000 per well in 96-well tissue culture coated plates, flat bottom, in the presence or absence of 80 μl of cisplatin at the concentrations indicated in the figure. After 72 h, 20 μl MTT reagent (Cell titer grow from Promega) were added, incubated for 30 min on a shaker and read on the BioTek Synergy Mx plate reader.

For spheroids, cells were plated at 2000 per well in 96-well ultralow round bottom attachment plates and incubated for 10 days in the presence of 50 μl of cisplatin at the concentrations indicated in the figure. After 10 days, 50 μl of Promega 3D were added, incubated for 30 min on a shaker and read using the BioTek Synergy Mx plate reader. Three independent experiments were performed and IC50 was obtained on prism and averaged.

### Ex vivo adhesion assay

This assay was performed as described earlier^13,58^. Briefly, ovaries from 16–17-days-old CD1 mice were wounded with a scalpel blade to mimic ovulation and each ovary was incubated with 30,000 fluorescently labeled cells (stably transduced with RFP lentiviral particles) overnight at 37 °C in an orbital shaker (40 rpm). The next day ovaries were washed several times, observable cells were counted, and representative pictures were taken with an AmScope MU900 with Toupview software (AmScope, Irvine, CA).

### Transfections

Pcmv-human-PAX2 was transfected into PTEN^shRNA^ or SCR^shRNA^ lines using Lipofectamine 2000 or TransIT LT1^TM^. Ten micrograms of plasmid per 10 cm dish was used and a ratio of Lipofectamine: plasmid of 3:1 in 1 ml total of optimem. The transfection media was then added to the cells in normal media and incubated for 48 h before processing. Single clones were isolated from PAX2-transfected PTEN^shRNA^ cells.

### Proliferation SRB assay

A total of 3000 cells were plated in triplicates in a clear flat–bottomed 96-well plate and allowed to attach overnight. Cells were incubated for 72 h at 37 °C and then fixed with 20% Trichloroacetic acid. Cell viability was determined using 0.04% Sulforhodamine B via colorimetric detection at 505. Normalization by time zero was performed and graph generated using GraphPad Prism software.

### Statistical analyses

All data are represented as mean ± standard error. Statistical analysis was carried out using Prism software (GraphPad, La Jolla, CA). All conditions were tested in three replicates in at least triplicate experiments. Statistical significance was determined by Student’s *t*-test when only two populations were compared; one-way ANOVA when more than two population were compared; two-ways ANOVA when more than two populations each divided into groups. *p ≤ 0.05 was considered significant.

## Supplementary information

Supplemental Figure 1

Supplemental figure 2

Supplemental figure 3

Supplemental figure legends

Sequences of primers used for qPCR

List of antibodies used for Western blot and IHC

## Data Availability

The data RNAseq analyzed in this study have been deposited to GEO: GSE157358. The other datasets are available from the correspondent author upon request.
