# Supplementary figures and images for: Silencing PTEN in the fallopian tube promotes enrichment of cancer stem cell-like function through loss of PAX2
Source: Cell Death Dis. 2021 Apr 7;12(4):375. doi: 10.1038/s41419-021-03663-2 (PMC8027874; doi:10.1038/s41419-021-03663-2)

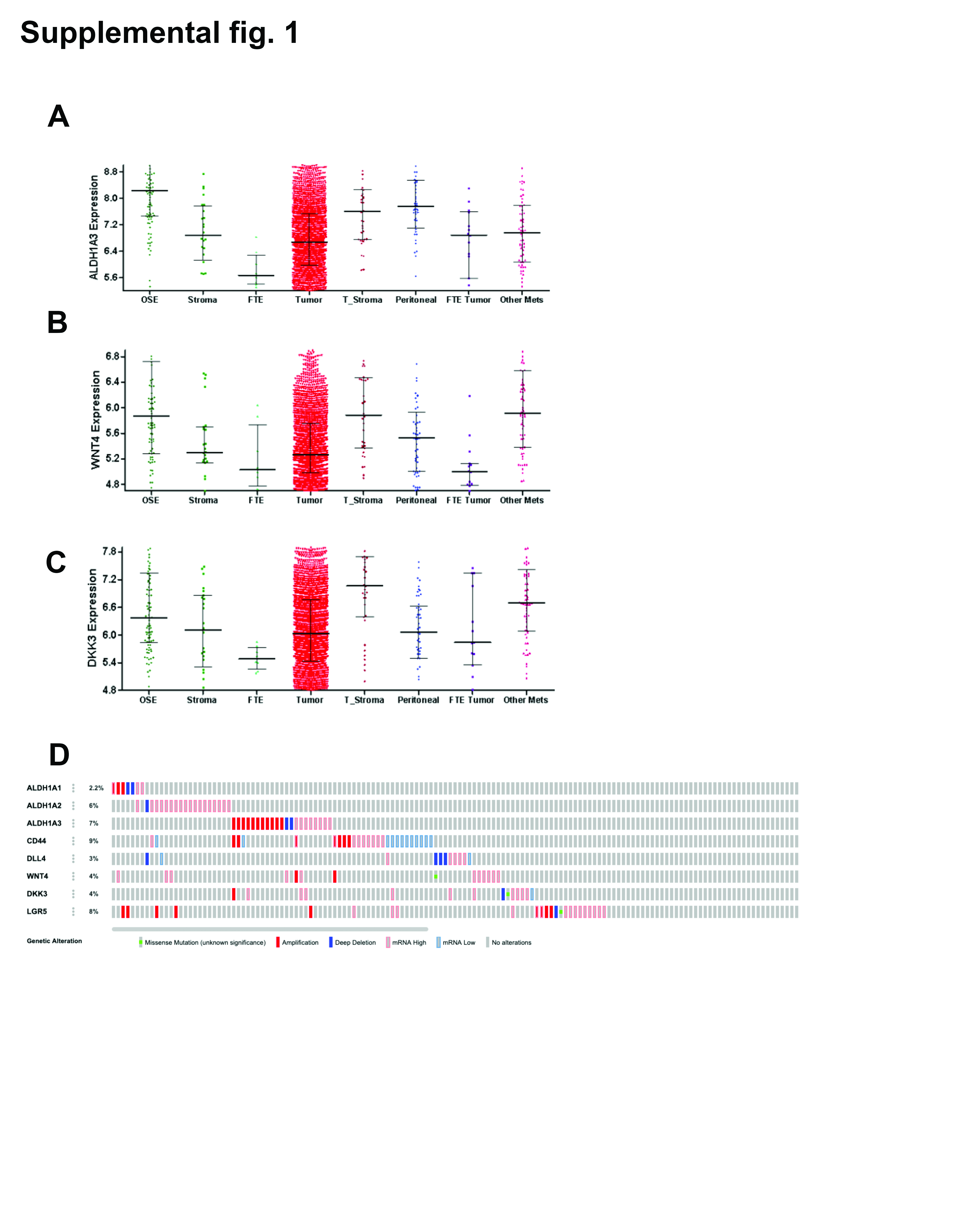

Supplement: Supplementary file 1 — Supplemental Figure 1 [file 41419_2021_3663_MOESM1_ESM.tif]

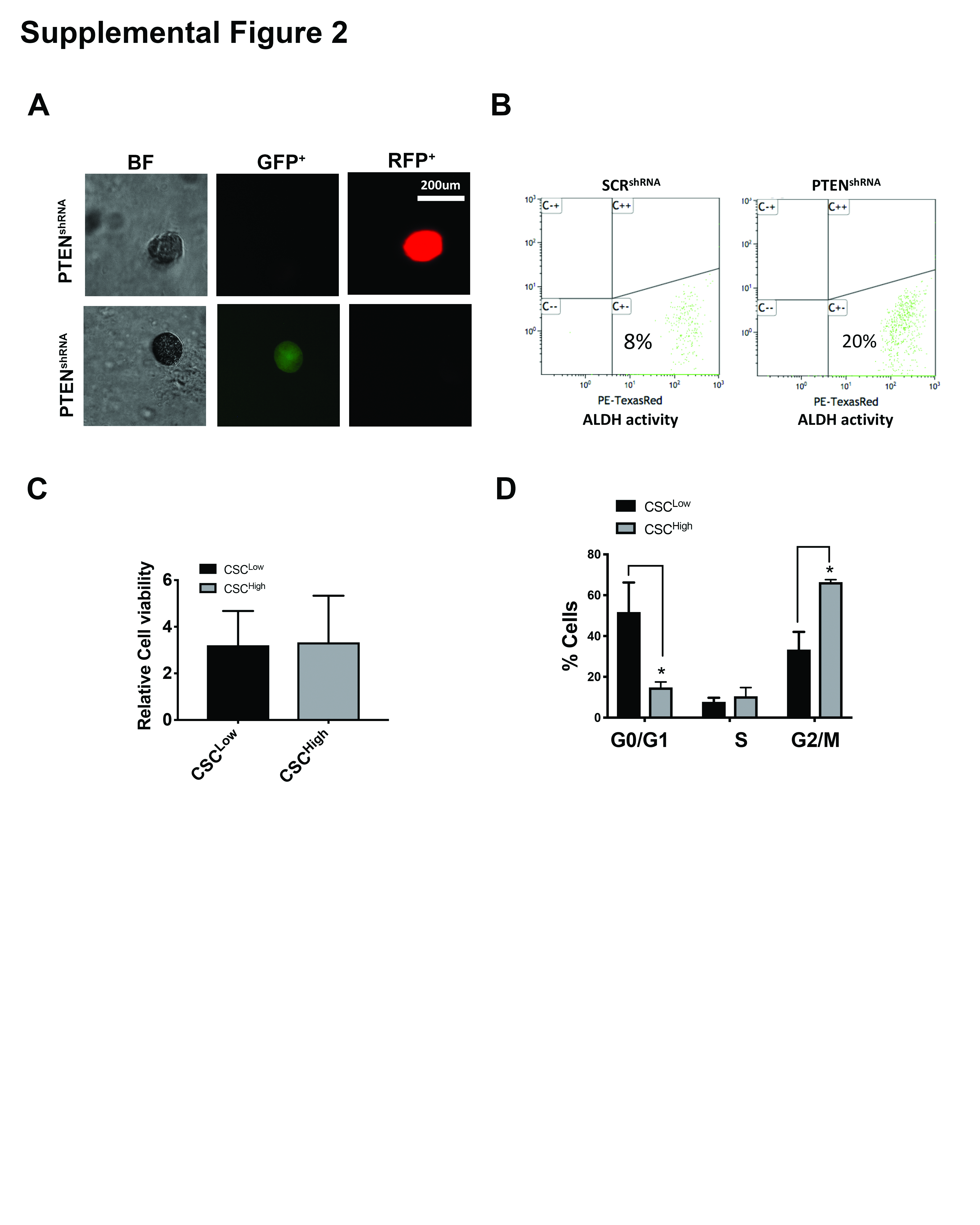

Supplement: Supplementary file 2 — Supplemental figure 2 [file 41419_2021_3663_MOESM2_ESM.tif]

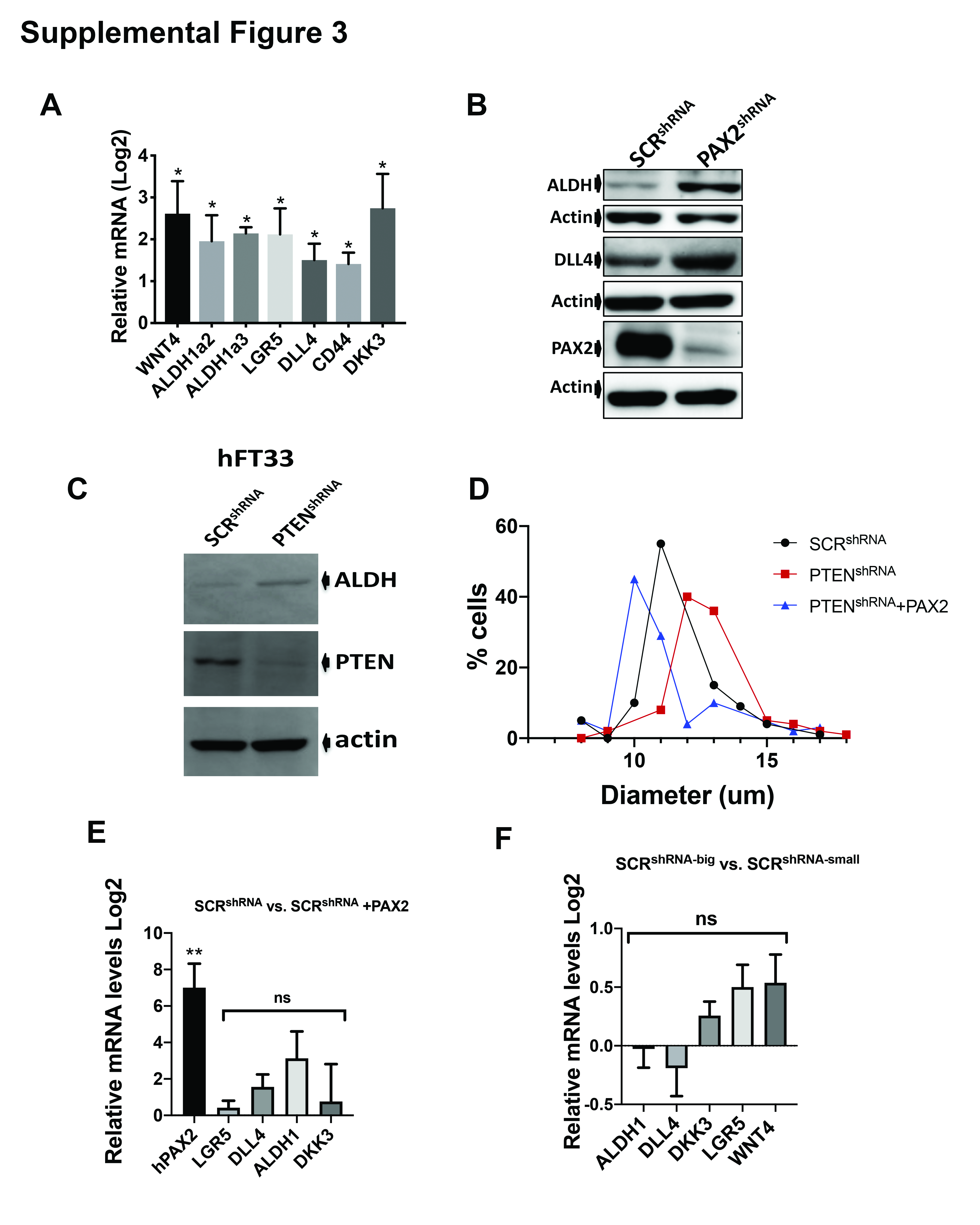

Supplement: Supplementary file 3 — Supplemental figure 3 [file 41419_2021_3663_MOESM3_ESM.tif]
