# Supplementary material for: Silencing PTEN in the fallopian tube promotes enrichment of cancer stem cell-like function through loss of PAX2
Source: Cell Death Dis. 2021 Apr 7;12(4):375. doi: 10.1038/s41419-021-03663-2 (PMC8027874; doi:10.1038/s41419-021-03663-2)
Supplement: Supplementary file 4 — Supplemental figure legends [file 41419_2021_3663_MOESM4_ESM.docx]

# Supplemental Figure legends

# Supplemental Figure 1. A-C) Singapore database analysis for ALDH1a3, WNT4 and DKK3 showing amplification in HGSOC as compared to human FTE. D) TCGA analysis showing amplification or mRNA up- regulation of CSC markers in HGSOC.

**Supplemental Figure 2. A)** PTEN^shRNA^ cells labeled with RFP or GFP were co-cultured in 50% Matrigel for 7 days and imaged using NIKON Eclipse TS100. Scale bar 200μm. **B)** ALDH activity was quantified using AldeRed 588 substrate and imaged using flowcytometry. **C)** Proliferation of CSC^Low^ vs. CSC^High^ was assessed for 5 days using SRB assay. **D)** Cell cycle analysis in CSC^Low^ vs. CSC^High^ using propidium iodide and analyzed using Cellometer K2 (Nexcelom).

**Supplemental Figure 3.** **A)** mRNA level of CSC markers in MOE cells expressing PAX2 shRNA vs. control. Three independent experiments were quantified, and statistical analysis was done using one-way ANOVA. **B)** Protein level of CSC markers in MOE cells expressing PAX2 shRNA vs. control. **C)** Human fallopian tube cells FT33 were targeted with PTEN^shRNA^ and analyzed for PTEN and ALDH expression by western blot. **D)** SCR^shRNA^, PTEN^shRNA^ and PTEN^shRNA^ plus PAX2 were trypsinized and diameter of cells in each population was quantified using Cellometer K2 from Nexcelom. **E)** SCR^shRNA^ MOE cells were transiently transfected with PAX2 plasmid and processed for qPCR. Three independent experiments were quantified, and statistical analysis was done using one-way ANOVA. **F)** SCR^shRNA^ MOE cells were subjected to cell sorting and two populations of cells were isolated based on size and processed for qPCR. Three independent experiments were quantified, and statistical analysis was done using one-way ANOVA.

# Supplemental Table I. Table with sequences of primers for qPCR.

# Supplemental Table II. Table with information relative to antibodies used for Western blot and IHC.
