# Supplementary material for: Silencing PTEN in the fallopian tube promotes enrichment of cancer stem cell-like function through loss of PAX2
Source: Cell Death Dis. 2021 Apr 7;12(4):375. doi: 10.1038/s41419-021-03663-2 (PMC8027874; doi:10.1038/s41419-021-03663-2)
Supplement: Supplementary file 5 — Sequences of primers used for qPCR [file 41419_2021_3663_MOESM5_ESM.pptx]

## Slide 1
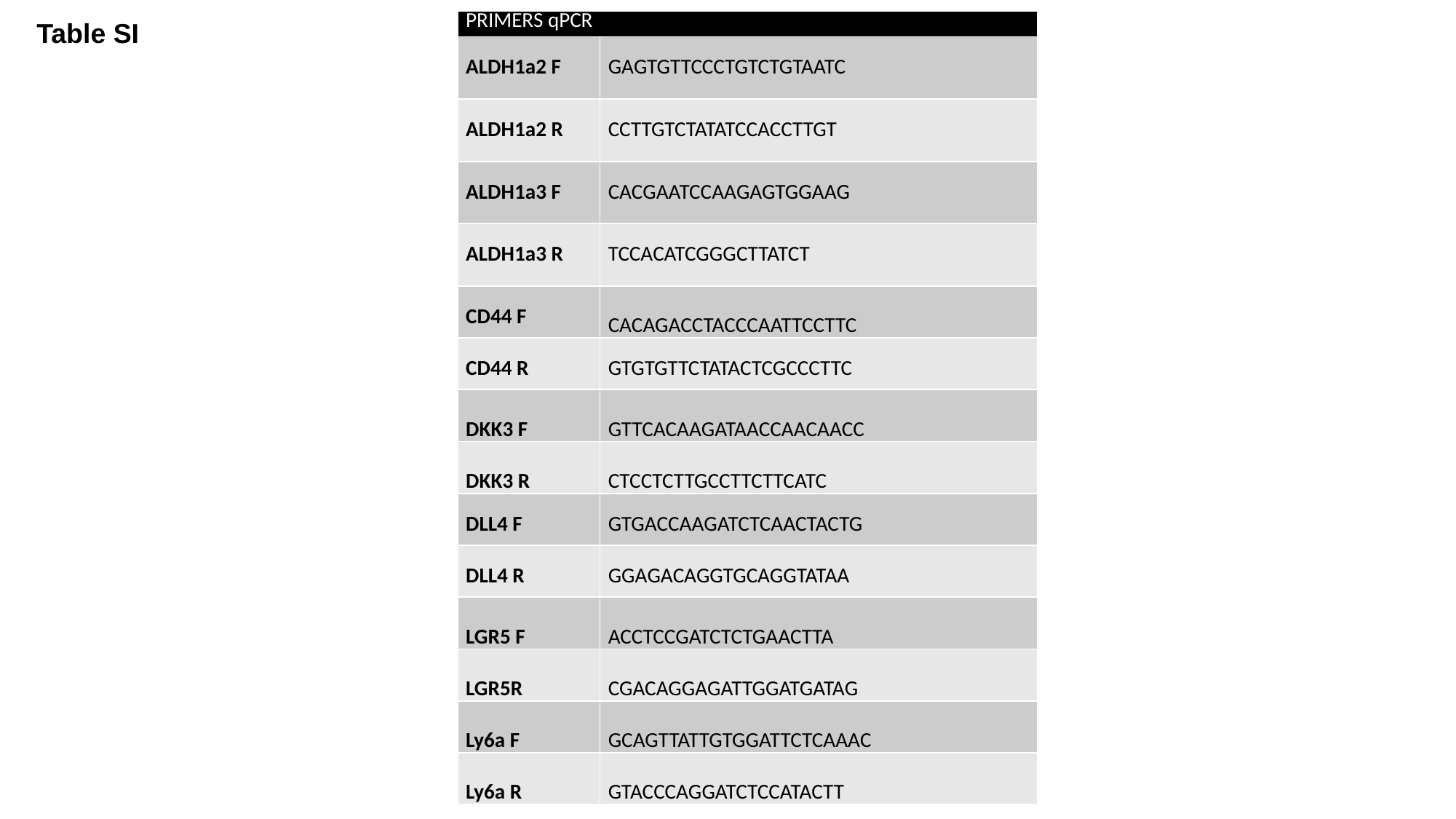

Table SI
| PRIMERS qPCR | |
| --- | --- |
| ALDH1a2 F | GAGTGTTCCCTGTCTGTAATC |
| ALDH1a2 R | CCTTGTCTATATCCACCTTGT |
| ALDH1a3 F | CACGAATCCAAGAGTGGAAG |
| ALDH1a3 R | TCCACATCGGGCTTATCT |
| CD44 F | CACAGACCTACCCAATTCCTTC |
| CD44 R | GTGTGTTCTATACTCGCCCTTC |
| DKK3 F | GTTCACAAGATAACCAACAACC |
| DKK3 R | CTCCTCTTGCCTTCTTCATC |
| DLL4 F | GTGACCAAGATCTCAACTACTG |
| DLL4 R | GGAGACAGGTGCAGGTATAA |
| LGR5 F | ACCTCCGATCTCTGAACTTA |
| LGR5R | CGACAGGAGATTGGATGATAG |
| Ly6a F | GCAGTTATTGTGGATTCTCAAAC |
| Ly6a R | GTACCCAGGATCTCCATACTT |
