# Supplementary material for: Silencing PTEN in the fallopian tube promotes enrichment of cancer stem cell-like function through loss of PAX2
Source: Cell Death Dis. 2021 Apr 7;12(4):375. doi: 10.1038/s41419-021-03663-2 (PMC8027874; doi:10.1038/s41419-021-03663-2)
Supplement: Supplementary file 6 — List of antibodies used for Western blot and IHC [file 41419_2021_3663_MOESM6_ESM.pptx]

## Slide 1
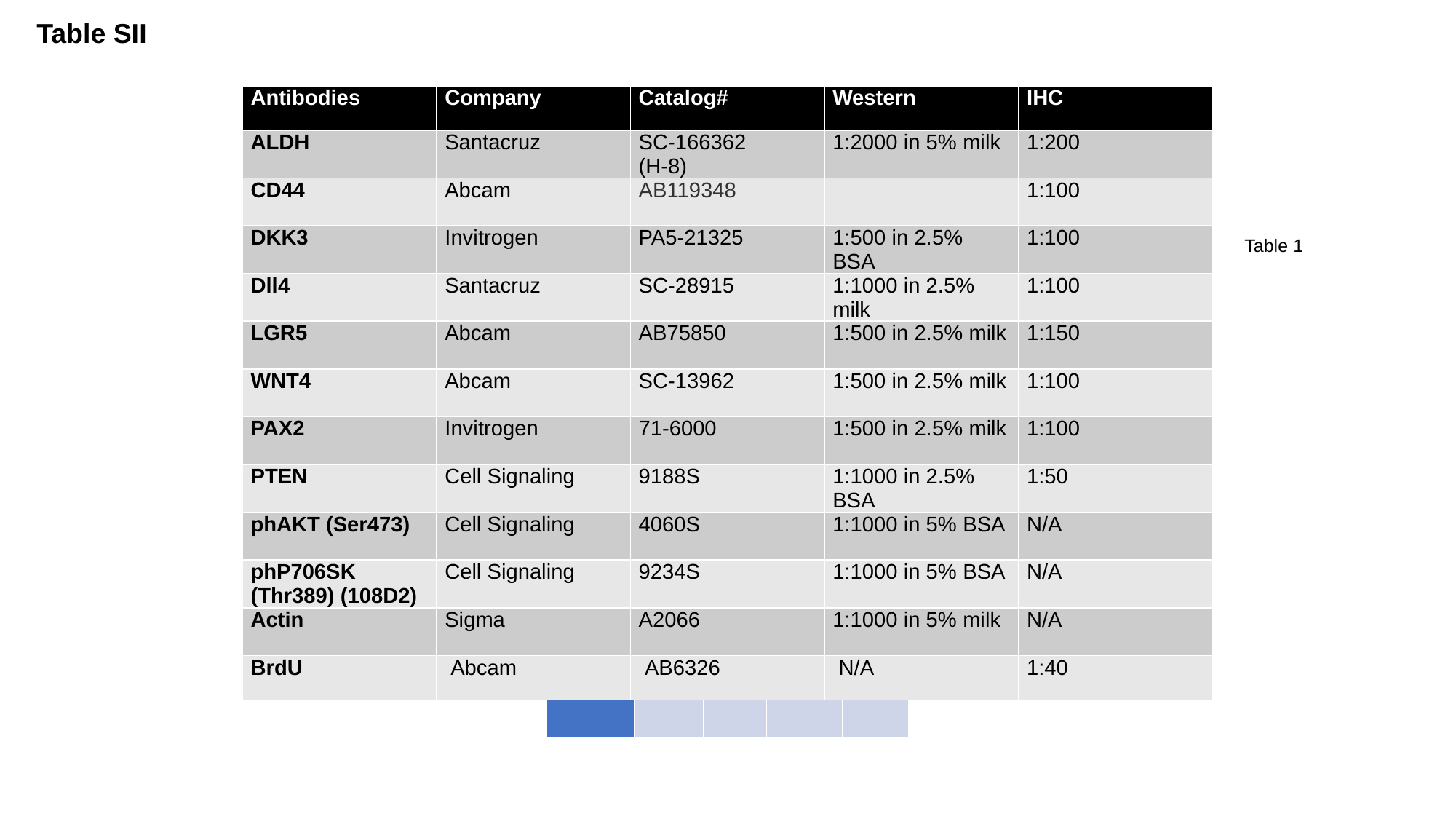

Table SII
| Antibodies | Company | Catalog# | Western | IHC |
| --- | --- | --- | --- | --- |
| ALDH | Santacruz | SC-166362 (H-8) | 1:2000 in 5% milk | 1:200 |
| CD44 | Abcam | AB119348 | | 1:100 |
| DKK3 | Invitrogen | PA5-21325 | 1:500 in 2.5% BSA | 1:100 |
| Dll4 | Santacruz | SC-28915 | 1:1000 in 2.5% milk | 1:100 |
| LGR5 | Abcam | AB75850 | 1:500 in 2.5% milk | 1:150 |
| WNT4 | Abcam | SC-13962 | 1:500 in 2.5% milk | 1:100 |
| PAX2 | Invitrogen | 71-6000 | 1:500 in 2.5% milk | 1:100 |
| PTEN | Cell Signaling | 9188S | 1:1000 in 2.5% BSA | 1:50 |
| phAKT (Ser473) | Cell Signaling | 4060S | 1:1000 in 5% BSA | N/A |
| phP706SK (Thr389) (108D2) | Cell Signaling | 9234S | 1:1000 in 5% BSA | N/A |
| Actin | Sigma | A2066 | 1:1000 in 5% milk | N/A |
| BrdU | Abcam | AB6326 | N/A | 1:40 |
| AB | Company | Catalog# | Western | IHC |
| --- | --- | --- | --- | --- |
| ALDH | Santacruz | SC-166362 (H-8) | 1:2000 in 5% milk | 1:200 |
| CD44 | Abcam | AB119348 | | 1:100 |
| DKK3 | Invitrogen | PA5-21325 | 1:500 in 2.5% BSA | 1:100 |
| Dll4 | Santacruz | SC-28915 | 1:1000 in 2.5% milk | 1:100 |
| LGR5 | Abcam | AB75850 | 1:500 in 2.5% milk | 1:150 |
| WNT4 | Abcam | SC-13962 | 1:500 in 2.5% milk | 1:100 |
| PAX2 | Invitrogen | 71-6000 | 1:500 in 2.5% milk | 1:100 |
| PTEN | Cell Signaling | 9188S | 1:1000 in 2.5% BSA | 1:50 |
| phAKT (Ser473) | Cell Signaling | 4060S | 1:1000 in 5% BSA | N/A |
| phP706SK (Thr389) (108D2) | Cell Signaling | 9234S | 1:1000 in 5% BSA | N/A |
| Actin | Sigma | A2066 | 1:1000 in 5% milk | N/A |
| BrDU | | | | 1:40 |
| | | | | |
Table 1
